# Emerging Health Disparities during the COVID-19 Pandemic

**DOI:** 10.1055/s-0042-1759842

**Published:** 2022-12-23

**Authors:** Nour Mheidly, Nadine Y. Fares, Mohamad Y. Fares, Jawad Fares

**Affiliations:** 1 Department of Communication, University of Illinois Chicago, Chicago, Illinois, United States; 2 Edinburgh Law School, University of Edinburgh, Edinburgh, United Kingdom; 3 Rothman Orthopaedic Institute, Philadelphia, Pennsylvania, United States; 4 Department of Neurological Surgery, Feinberg School of Medicine, Northwestern University, Chicago, Illinois, United States; 5 School of Information Technology, York University, Toronto, Ontario, Canada

**Keywords:** COVID-19, health equity, disparities, minorities, United States

## Abstract

The coronavirus disease 2019 (COVID-19) pandemic has underscored social and racial discrimination in global health, showing that health equity is still a goal to be achieved. Understanding the impact of COVID-19 on public health potential is vital to present a fair opportunity for people of different backgrounds to be as healthy as possible. As such, this communication discusses the emerging health disparities in light of the COVID-19 pandemic and analyzes their implications. Original research, effective health communication, and promotion strategies ought to be leveraged to step closer toward national and international health equity.

## Introduction

**Fig. 1:**
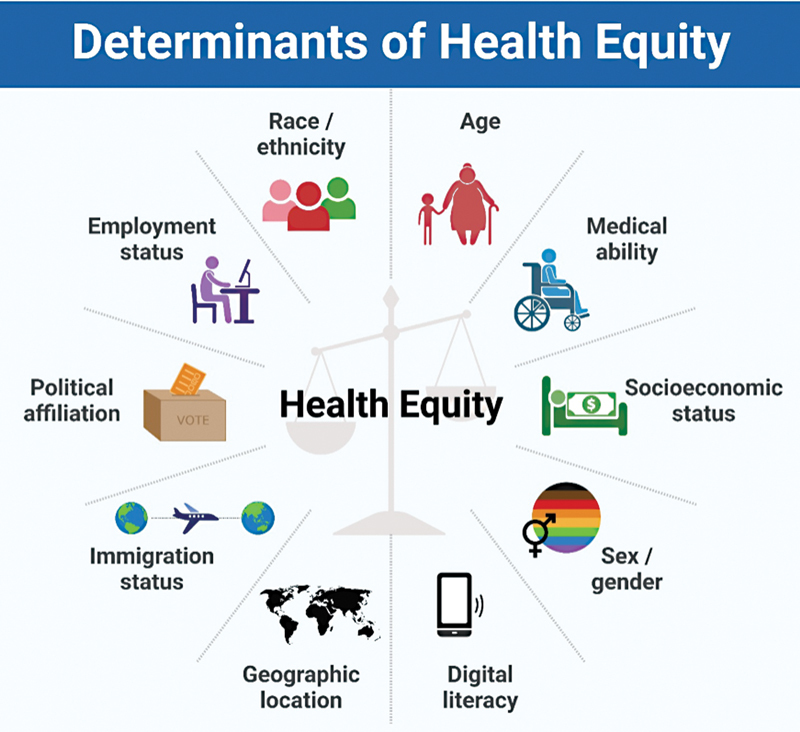
Emerging determinants of health equity during the coronavirus disease 2019 pandemic.

Health inequities and differences reflect the social and demographic order of the world and the wide history of racialized behavior and colonialism. 1 The coronavirus disease 2019 (COVID-19) pandemic highlighted disparities in the social determinants of health that existed worldwide **( Fig. 1 )** . Health inequity increased the risk of people of certain backgrounds of getting ill or dying from the disease. Factors that contributed to the increased risk included lack of testing sites, medical care, essential resources, and the existing chronic medical conditions among underserved populations. 2 In this article, we explore the emerging health equity themes in light of the COVID-19 pandemic and analyze their implications to improve health outcomes in the future.

## Health Inequity during the COVID-19 Pandemic

Race and ethnic backgrounds were important factors that contributed to health inequity during the pandemic. In the United States, the mortality and morbidity due to COVID-19 were higher among the Black communities, Latinx, Asian Americans, Pacific Islanders, and other vulnerable populations. 3 4 According to the Centers for Disease Control and Prevention, COVID-19 cases were 1.5 times higher among Native Americans, Alaska natives, and Hispanics in comparison to Whites. 5 COVID-19 hospitalization was close to three times higher in Native Americans and Alaska natives, and two times higher in Hispanics and Blacks than Whites. 5 COVID-19 deaths were approximately two times higher in Native Americans, Alaska natives, Hispanics, and Blacks in comparison to Whites. 5 The ethnic and racial discrepancies in COVID-19 morbidity and mortality were due to high exposure to the severe acute respiratory syndrome coronavirus 2 (SARS-CoV-2) virus associated with socioeconomic status and living conditions, and former medical conditions that amplify the risks of severe COVID-19 disease in ethnic and racial minority groups. During the period of March to December 2020, a database of approximately 300,000 hospitalized COVID-19 patients showed that groups of racial and ethnic minorities, such as Hispanic and Black patients, had the highest risks of infection and odds of hospitalization in comparison to White patients. 6 Even in the British medical reports, Asian patients comprised the highest odds of hospitalization, stressing on the unequal and disproportionate impact that this virus had on multiple communities. 7

A good number of deaths assigned to COVID-19 were in fact ascribed to factors other than the virus itself, such as sociodemographic and health characteristics, race/ethnicity, sex, and gender. In the United States, stratified models were examined to show that these factors were substantially evident in communities with low household incomes, inadequate education, and poorer health systems. 8 COVID-19 mortality was five times higher for adults in low socioeconomic positions than for those in high socioeconomic positions (72.2 vs. 14.6 deaths per 100000). Being of low socioeconomic status, Hispanic ethnicity and male gender carried a COVID-19 mortality risk that was 27 times higher. COVID-19 mortality risk was lowest for White women with high socioeconomic standing (6.5 deaths per 1,00,000). 9

With the COVID-19 economic fallout, the stress and fear of financial insecurity were most detrimental among socially disadvantaged groups. For example, data from the United States Bureau of Labor Statistics indicate that, between April 2019 and April 2020, the unemployment rate in the United States increased from 3.6 to 14.7%, but from 11.5 to 31.2% and 3.7 to 16.7% particularly among the Black and Latinx communities, respectively. 10 To maintain financial security, Black and Hispanic individuals were more likely to work, and for a longer term, in high-COVID-19 risk occupations like healthcare and transportation, 11 increasing exposure to SARS-CoV-2 and the likelihood of infection.

Due to social and political factors, im/migrant populations, the LGBTQ+ community, and intersecting individuals suffered from social vulnerability and thus had to tolerate health inequity along with stigmatized perceptions throughout the pandemic. Unstable immigration statuses can lead to im/migrants engaging in work fields of substantial health risks without employer-provided health insurance. Consequently, they have higher risks of exposure to COVID-19 compared with residential employees. 12 Like immigration status, sexual orientation and gender identity were a source of social vulnerability. Populations of the LGBTQ+ community have been defamed during past outbreaks of novel transmissible diseases such as “AIDS, severe acute respiratory syndrome (SARS), and H1N1.” This leads to discriminatory communication and inadequate COVID-19 treatment of these groups. 12

Age played a factor in the impact of the virus, where there was evidence that adults had an increased risk of acquiring SARS-CoV-2 compared with children. 13 Older adults reported higher depression and greater loneliness following the onset of the pandemic. 14 Racial migrant workers were also dramatically impacted by the lockdowns. Their financial situation precluded their ability to get access to medical care. They were also unable to adhere appropriately to social distancing, quarantine, or lockdowns due to the absence of food and medical aid, which put them in direct danger of infection. 15 Moreover, patients who were aged over 65 years were reported less likely to use video services in telehealth care. 16

In aims of providing services more efficiently, many health systems switched to telehealth and fast technological solutions. This change, however, risked intensifying health inequity among racial and socioeconomic groups. In a single-institutional study in the United States, Black and White patients had more access to telehealth care compared with Hispanic and Asian patients. 16 Furthermore, White patients were more likely to utilize video technology services than Black, Asian, and Hispanic patients. 16 People with deficient technological resources and poor digital literacy could not access digital health as an alternative solution for health delivery during lockdowns and social distancing. 17 18 This put them at risk of not having their communicable and noncommunicable diseases managed properly.

The vaccine rollout was another demonstration of the disparities that existed among populations. In December 2020, in the first month of vaccination, among 6,706,697 persons in the United States, 60.4% were White, and 39.6% were ethnic minorities. 17

Politics played an important role in influencing health beliefs and actions during the COVID-19 pandemic. 19 In the United States, when the public looked for governmental leadership to satisfy their need for information, they found COVID-19 to be a topic of political debate among opposing political parties. This uncertainty fueled conspiracy theories and misinformation, which led to fear and anxiety among the public. 19 Politics during the pandemic lacked scientific merit when tackling COVID-19, which negatively affected public health outcomes. In Brazil, supporters of conservative politicians that undermined protective measures had the greatest number of COVID-19 mortalities in 2021. 20 Globally, dominant industrial nations, such as the United States and China, prioritized the well-being of their citizens by providing them exclusively with resources. 21 For example, the US government requested firms, such as “3M,” to only manufacture face masks for the nation, and to refrain from sending masks to Latin America or Canada. 21

## Recommendations and Insights for the Future

A multifaceted approach must be taken when promoting health equity during pandemics. This approach should ensure the availability of trusted health information, accessibility to resources and infrastructure, and proper engagement with members of the community. 22 It is paramount that health information resources in a community adhere to proper standards of quality and evidence. 23 The presence of fallacious references can hinder public health awareness, and this was especially evident during the COVID-19 pandemic; where, due to the scarcity of properly available trusted medical sources, an “infodemic” arose. 23 Widespread transmission of medical misinformation and erroneous guidelines took place on social media, and many communities with no access to trustworthy and evidence-based resources were rendered misinformed. 23 Accordingly, governing bodies and authorities should provide easily available platforms to spread health information and answer any queries from members of the community. 22 23 This has been implemented in some countries, where COVID-19 hotlines had been initiated to tackle any questions or issues regarding the pandemic from concerned citizens of all backgrounds. 24 25 26

It is also essential to ensure the availability of proper infrastructure and resources that can accommodate health information platforms and services. 22 This is especially important in marginalized communities, where access to electricity and Internet is not as easy. 27 27 29 The pandemic prompted a movement toward telemedicine and online consultations, and many people did not have access to technologies that allow the usage of such tools. 30 This problem can be managed by ensuring Internet and electricity coverage throughout all marginalized communities and providing stimulus packages to suffering families that cannot afford the needed tools and devices for such services. In addition, adequate and targeted public health campaigns that aim to increase health media literacy should be supported and popularized in marginalized communities.

As with any other public health intervention, it is important to properly engage with the communities undertaking a health-related intervention, for example, virtual healthcare during a pandemic. 22 That is particularly important with marginalized populations that are less likely to adapt and integrate these novel changes and regulations to their lives. 31 Surveying communities and requesting feedback are essential for exploring any deficits, noting any complaints, and reporting any economic, linguistic, or cultural issues of certain subpopulations. 22 32 In this way, governing bodies and authorities would be able to directly pinpoint and explore disparities that can lead to health inequities and resolve them.

## Conclusion

The COVID-19 pandemic highlighted emerging inequalities in health. Funding research endeavors that aim to understand reasons behind inequalities in health and improve the current status quo is important to bridge the gaps in health worldwide. Health communication and promotion strategies aimed at people of different backgrounds and beliefs are needed to step closer toward global health equity. 23
